# The calcium-sensing receptor suppresses epithelial-to-mesenchymal transition and stem cell- like phenotype in the colon

**DOI:** 10.1186/s12943-015-0330-4

**Published:** 2015-03-18

**Authors:** Abhishek Aggarwal, Maximilian Prinz-Wohlgenannt, Charlotte Gröschel, Samawansha Tennakoon, Anastasia Meshcheryakova, Wenhan Chang, Edward M Brown, Diana Mechtcheriakova, Enikö Kállay

**Affiliations:** Department of Pathophysiology and Allergy Research, Medical University of Vienna, Währinger Gürtel 18-20, A-1090 Vienna, Austria; Endocrine Research Unit, Department of Veteran Affairs Medical Center, University of California, San Francisco, CA USA; Department of Medicine, Division of Endocrinology, Diabetes and Hypertension, Brigham and Women’s Hospital, Boston, MA USA

**Keywords:** Calcium-sensing receptor, Tumor suppressor, Colon, Inflammation, Cancer, EMT, Cancer stem cell, Calcimimetic

## Abstract

**Background:**

The calcium sensing receptor (CaSR), a calcium-binding G protein-coupled receptor is expressed also in tissues not directly involved in calcium homeostasis like the colon. We have previously reported that CaSR expression is down-regulated in colorectal cancer (CRC) and that loss of CaSR provides growth advantage to transformed cells. However, detailed mechanisms underlying these processes are largely unknown.

**Methods and results:**

In a cohort of 111 CRC patients, we found significant inverse correlation between CaSR expression and markers of epithelial-to-mesenchymal transition (EMT), a process involved in tumor development in CRC. The colon of *CaSR*/*PTH* double-knockout, as well as the intestine-specific *CaSR* knockout mice showed significantly increased expression of markers involved in the EMT process. *In vitro*, stable expression of the CaSR (HT29^CaSR^) gave a more epithelial-like morphology to HT29 colon cancer cells with increased levels of E-Cadherin compared with control cells (HT29^EMP^). The HT29^CaSR^ cells had reduced invasive potential, which was attributed to the inhibition of the Wnt/β-catenin pathway as measured by a decrease in nuclear translocation of β-catenin and transcriptional regulation of genes like GSK-3β and Cyclin D1. Expression of a spectrum of different mesenchymal markers was significantly down-regulated in HT29^CaSR^ cells. The CaSR was able to block upregulation of mesenchymal markers even in an EMT-inducing environment. Moreover, overexpression of the CaSR led to down-regulation of stem cell-like phenotype.

**Conclusions:**

The results from this study demonstrate that the CaSR inhibits epithelial-to-mesenchymal transition and the acquisition of a stem cell-like phenotype in the colon of mice lacking the CaSR as well as colorectal cancer cells, identifying the CaSR as a key molecule in preventing tumor progression. Our results support the rationale to develop new strategies either preventing CaSR loss or reversing its silencing.

**Electronic supplementary material:**

The online version of this article (doi:10.1186/s12943-015-0330-4) contains supplementary material, which is available to authorized users.

## Background

Colorectal cancer (CRC) is the second most frequently diagnosed malignant tumor in females, third most in males, and ranks second in cancer related deaths worldwide [1,2]. Chronic inflammation is one of the major risk factors to develop colorectal tumors [3]. Patients with inflammatory bowel disease have an increased risk to develop cancer [4,5]. Although progress in therapy to manage locally advanced or metastatic CRC patients have evolved, the 5-year survival rate continues to be poor (www.cancer.org).

Epithelial-to-Mesenchymal Transition (EMT) is a reversible, cellular trans-differentiation process by virtue of which epithelial cells acquire mesenchymal traits of plasticity, mobility, stem cell-like and invasive properties [6]. The EMT process, which is normally active during embryogenesis and wound healing (Type 1 EMT), can be activated also during tissue regeneration and fibrosis upon initiation by inflammation (Type 2 EMT) [7]. Type 3 EMT occurs during tumor progression when cancer cells exploit this mechanism to gain invasive and metastatic potential [7,8]. The reversal of this process, called Mesenchymal-to-Epithelial Transition (MET), is implicated in later stages of metastasis by which metastatic cancer cells reacquire epithelial characteristics in order to form secondary tumors [9].

Activation of the EMT program orchestrates complex transformations in cellular architecture and behavior. A spectrum of EMT regulators, majority of which belong to the zinc-finger family of transcription factors (including, but not restricted to Snai1 (Snail), Snai2 (Slug), Twist, and Zeb) can bind to the promoter of E-Cadherin and repress its transcription [6,10]. Downregulation of E-Cadherin results in loss of the epithelial phenotype (by deregulated inter-cellular junction complexes), accompanied by the induction of genes specific for mesenchymal phenotype (e.g. αSMA, FSP1, FOXC2 and Vimentin). Furthermore, reduction of E-Cadherin-dependent sequestering of cytoplasmic β-catenin results in free β-catenin that is able to translocate to the nucleus and activate the Wnt/β-catenin signaling pathway [11]. These cells also undergo a ‘cadherin-switch’ leading to a shift from E-Cadherin to N-Cadherin expression, which is operated by the aforementioned epithelial transcriptional repressors [10,12].

In solid tumors, including CRC, recent reports have shown that a small sub-population of cells, within the heterogeneous tumor, acquires features of stem cells [13,14]. These cancer stem cells have the ability of self-renewal and usually increase in number following conventional therapy as these treatments target only the rapidly dividing cells.

The calcium-sensing receptor (CaSR) is a G protein-coupled receptor that regulates systemic calcium homeostasis. In the colon, the CaSR has been shown to play important roles in nutrient sensing and fluid secretion/absorption [15]. CaSR expression is significantly reduced in colorectal tumors [16,17]. Since the chemopreventive properties of Ca^2+^ are partially mediated by the CaSR [18,19], these effects may be limited in colonic tumors lacking the CaSR. Indeed, the colons of mice lacking the CaSR exhibit aberrant crypt foci, the earliest identifiable pre-neoplastic lesions [20] as well as enhanced intestinal inflammation [20,21] suggesting that the CaSR is important for maintaining normal colonic epithelium.

In this study we aimed to decipher the mechanisms that contribute to the tumor suppressive functions of the CaSR during colorectal tumorigenesis. We demonstrate that the CaSR suppresses EMT and the stem cell-like phenotype both, *in vivo* in the colon of mice and *in vitro*, in colon cancer cells, providing rationale for developing pharmacological agents to modulate CaSR sensitivity in colorectal cancer to prevent tumor progression.

## Results

### Ablation of CaSR leads to induction of EMT-associated markers in the colon of global CaSR/PTH double-knock out mice

We investigated the consequences of CaSR knockdown on markers of EMT in the colon of two murine models of *CaSR* gene knockdown.

**Figure 1:**
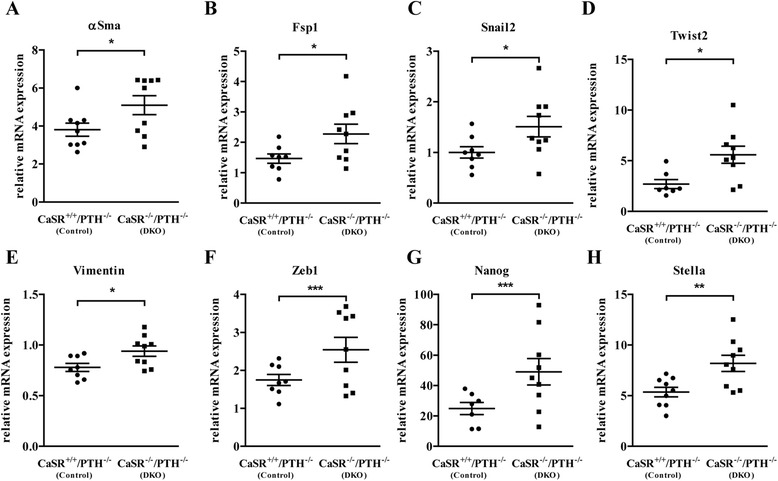
**Effect of global CaSR knock-down** ***in vivo*** **on genes modulating EMT/stemness in the colon.** Colons sampled from *CaSR* ^−/−^/*PTH* ^−/−^ (DKO) and *CaSR* ^+/+^/*PTH* ^−/−^ (control) mice were investigated for mRNA expression of the mesenchymal markers αSma **(A)**, Fsp1**(B)**, Snail2 **(C)**, Twist2 **(D)**, Vimentin **(E)**, Zeb1 **(F),** and the pluripotency markers Nanog **(G)** and Stella **(H)**. Dots indicate individual data points, and the line represents median. Statistical significance was calculated using t test. n = 9 animals/group, *p < 0.05, **p < 0.01, ***p < 0.001.

In the first model, global ablation of exon-5 of the CaSR on a PTH-null background (*CaSR*^−/−^/*PTH*^−/−^, DKO) led to a significant upregulation in mRNA expression of the mesenchymal markers αSma, Fsp1, Snai2, Twist2, Vimentin and Zeb1 in the colon compared with control mice (*CaSR*^+/+^/*PTH*^−/−^) (Figure 1). Expression of the pluripotency markers, Nanog and Stella, was also significantly elevated in the colon of DKO mice compared with controls (Figure 1).

### Ablation of CaSR leads to induction of EMT-associated markers in the colon of intestine-specific CaSR knock out mice

**Figure 2:**
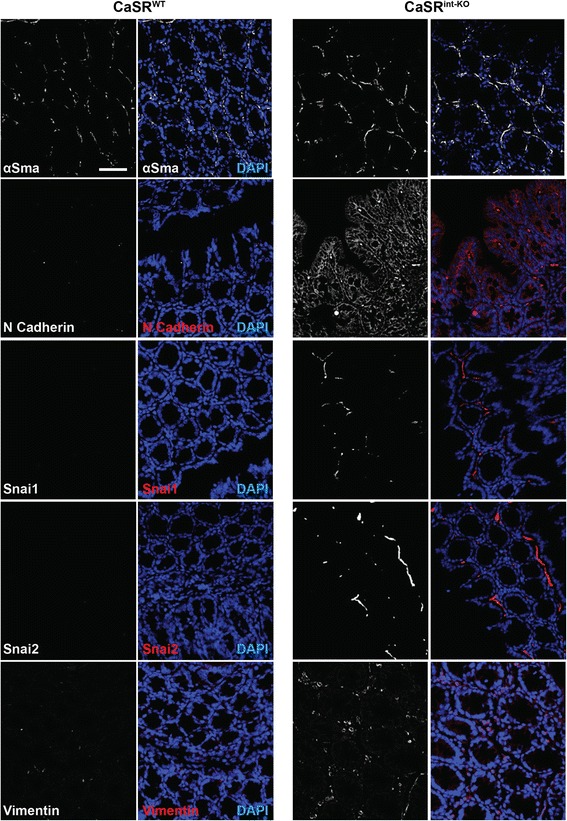
**Effect of intestine-specific CaSR knock-down** ***in vivo*** **on EMT-associated genes in the colon.** Colons sampled from *CaSR* ^−/−^ (CaSR^int-KO^) and *CaSR* ^+/+^ (CaSR^WT^) mice were investigated for protein expression of mesenchymal markers αSma, N-cad, Snai1, Snai2 and Vimentin. Representative images in black and white for the markers are shown in addition to the merged images (red or white channels for the indicated markers and blue for DAPI). n = 5 animals/group. Scale bar: 50 μm.

We further evaluated protein expression of EMT markers in colon of mice lacking exon-7 of the CaSR specifically in the intestinal epithelium under the control of the Villin promoter (CaSR^int-KO^). Colonocytes lacking CaSR in the intestinal epithelia showed a clear upregulation in expression of N-Cadherin (in the epithelial cells of the crypt) and of αSma and Vimentin (in the stromal region) compared with wild-type mice (CaSR^WT^, Figure 2). Although N-Cadherin expression was upregulated in CaSR^int-KO^ mice, expression of E-Cadherin was unaltered compared with CaSR^WT^ mice (data not shown). Interestingly, we also saw populations of Snai1 and Snai2 expressing cells limited to the stroma only in the CaSR^int-KO^ mice (Figure 2).

### Overexpression of the CaSR enhances the epithelial phenotype of HT29 colon cancer cells

**Figure 3:**
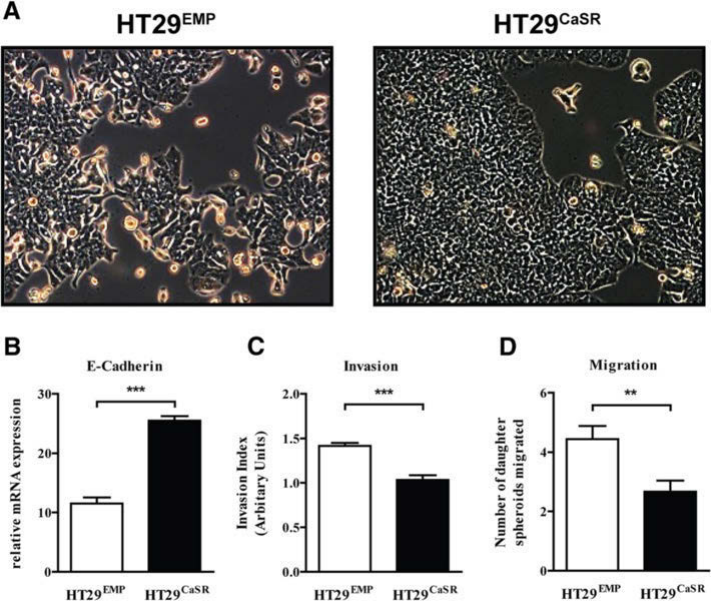
**Impact of the CaSR on the invasive potential of HT29 colon cancer cells. (A)** HT29^EMP^ cells have an elongated, spindle-like morphology whereas HT29^CaSR^ cells attain a more compact, epithelial-like morphology of well adherent cells. **(B)** Expression of the epithelial marker E-Cadherin is significantly upregulated in HT29^CaSR^ cells compared with HT29^EMP^ cells. **(C-D)** Invasion and migration was assessed using a 3D spheroid forming assay. The area of the spheroid invading the surrounding matrix is presented as the invasive index. The number of daughter spheroids migrated from the parental spheroid was counted. Bars represent means ± SEM of three independent experiments. Statistical significance was calculated using t tests. **p < 0.01, ***p < 0.001.

Although of epithelial origin, HT29 colon cancer cells grow as spindle-shaped, elongated cells that contact neighboring cells only focally when cultured in their standard growth conditions. While HT29^EMP^ cells retained this mesenchymal-like phenotype, stable transfection with the CaSR promoted a morphological change in these cells to a more cobblestone-like, well adherent phenotype that displayed complete cell-cell adhesion with the cells growing in densely packed colonies (Figure 3A). Furthermore, mRNA expression of the epithelial marker, E-Cadherin was significantly upregulated in HT29^CaSR^ compared with HT29^EMP^ cells (Figure 3B).

### Overexpression of the CaSR impairs migration and invasion of colon cancer cells

Since tumor cell spheroids are considered more representative of *in vivo* conditions, we evaluated the role of the CaSR in regulating migration and invasion of CRC cells in a 3D spheroid cell invasion assay. After spheroid formation for 7 days, the migration and invasion potential of 3D cellular aggregates into the surrounding matrix was evaluated.

HT29^CaSR^ cells had significantly lower invasive index (area of the invading spheroids) compared with cells that were transfected with the empty vector (Figure 3C). To distinguish between effects on migration and invasion, we additionally quantified the number of daughter spheroids that had migrated away from the primary spheroid. Overexpression of the CaSR significantly reduced the number of invading daughter spheroids compared with control cells (Figure 3D).

### Overexpression of the CaSR attenuates nuclear translocation of β-catenin in HT29 colon cancer cells

**Figure 4:**
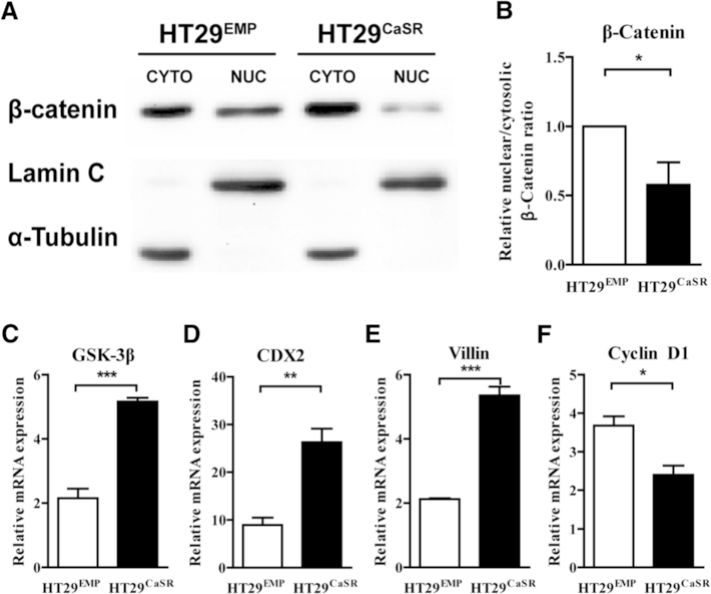
**Ectopic CaSR prevents nuclear β-catenin translocation in HT29 colon cancer cells. (A)** HT29 cells overexpressing the CaSR (HT29^CaSR^) show reduced β-catenin nuclear translocation as assessed by western blot and **(B)** by quantification of β-catenin signal normalized to house-keeping genes (Lamin C: nuclear fraction and α-Tubulin: cytosolic fraction). **(C-F)** HT29^CaSR^ cells show increased mRNA expression of GSK-3β, of the differentiation markers CDX2 and Villin, and reduced levels of the proliferation marker Cyclin D1 compared with HT29^EMP^ cells. Data represent mean ± SEM of three independent experiments. Statistical significance was calculated using t test. *p < 0.05, **p < 0.01, ***p < 0.001.

Previous studies have shown that loss of CaSR promotes migration and invasion of CRC cells by regulating the Wnt/β-catenin pathway [20,22,23]. Since ectopic CaSR enhanced the epithelial phenotype whilst inhibiting the invasiveness of HT29 cells, we examined whether restoration of CaSR expression was indeed able to regulate Wnt/β-catenin activity. We measured β-catenin expression in protein lysates from nuclear and cytosolic fractions of HT29^EMP^ and HT29^CaSR^ cells. Cells overexpressing the CaSR had a marked decrease in the amount of nuclear β-catenin (Figure 4A). The ratio of nuclear to cytosolic β-catenin in HT29^CaSR^ cells was significantly decreased by 43% compared with HT29^EMP^ cells (Figure 4B). Concomitantly we found significantly higher GSK-3β mRNA expression in these cells (Figure 4C).

We showed that overexpression of CaSR increased expression of the differentiation markers, CDX2 and Villin (Figure 4D and E), and downregulated expression of the proliferation marker, Cyclin D1 (Figure 4F).

### CaSR suppresses EMT in HT29 colon cancer cells

**Figure 5:**
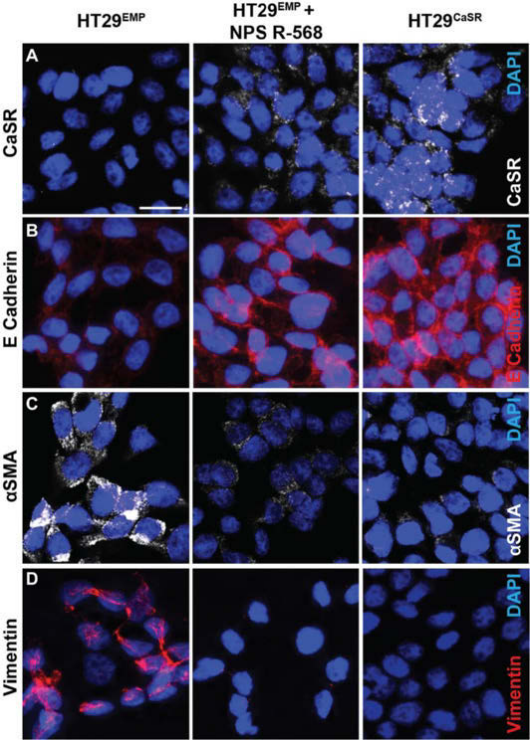
**Induction of CaSR expression/function suppresses EMT in HT29 colon cancer cells.** Expression of CaSR and E-Cadherin are upregulated in HT29^EMP^ cells treated with 1 μM NPS R-568 or in HT29^CaSR^ cells whereas expression of αSMA and Vimentin is downregulated. The merged images (red or white channels for the indicated markers and blue for DAPI) are shown. Scale bar: 20 μm.

NPS R-568, a positive allosteric modulator of the CaSR increases sensitivity of the receptor to its ligands, including Ca^2+^ [24]. Interestingly, treatment with NPS R-568 upregulated the endogenous expression of the CaSR in HT29^EMP^ cells (Figure 5A). Both, the ectopic (HT29^CaSR^) and the endogenous CaSR (HT29^EMP^ treated with NPS R-568) were able to induce expression of E-Cadherin (distinctively in the cell membrane) (Figure 5B) and down-regulate the expression of the mesenchymal markers such as αSMA and Vimentin (Figure 5C and D).

**Figure 6:**
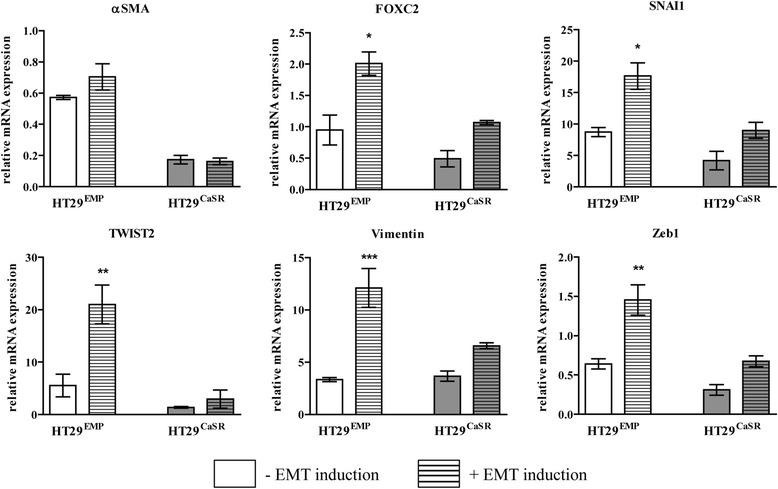
**Ectopic CaSR prevents induction of mRNA expression of EMT markers in HT29 colon cancer cells.** HT29^CaSR^ cells (grey bars) show downregulation in mRNA expression of mesenchymal markers αSMA, FOXC2, SNAI1, TWIST2, Vimentin and Zeb1 compared with HT29^EMP^ cells (white bars). Treatment with EMT promoting cocktail, further induced mesenchymal transition in HT29^EMP^ cells (white striped bars), which was blocked by ectopic expression of CaSR (HT29^CaSR^, grey striped bars). Data represent mean ± SEM of three independent experiments. Statistical significance was determined by ANOVA followed by Tukey’s post-test. Asterisks above bars indicate all significant changes. *p < 0.05, **p < 0.01, ***p < 0.001.

We next evaluated whether the presence of the CaSR would further prevent induction of EMT in HT29 cells. Stably transfected HT29 cells were treated with a commercially available EMT inducing cocktail. Upon treatment, HT29^EMP^ cells were robustly induced towards the mesenchymal phenotype as assessed by significant upregulation in mRNA expression of the mesenchymal markers αSMA, FOXC2, SNAI1, TWIST2, Vimentin and Zeb1 (Figure 6). Interestingly, in HT29^CaSR^ cells, ectopic reintroduction of the CaSR was able to block EMT induction in these cells (Figure 6).

**Figure 7:**
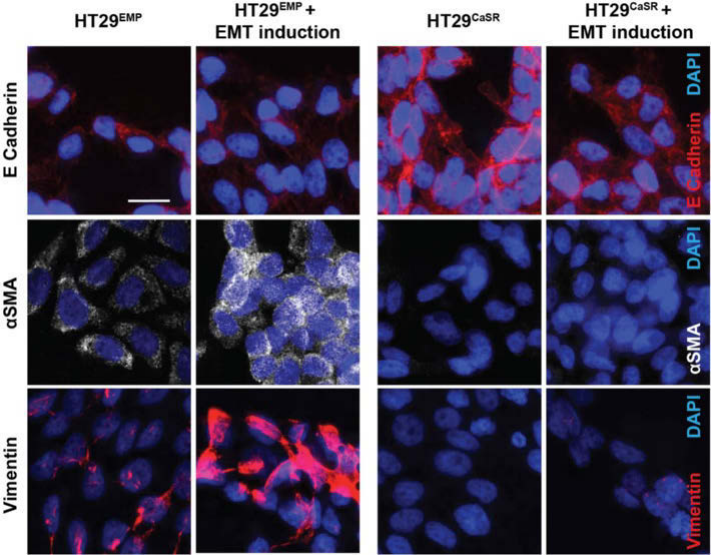
**Ectopic CaSR prevents induction of protein expression of EMT markers in HT29 colon cancer cells.** Treatment with the EMT promoting cocktail induced protein expression of mesenchymal markers, αSMA and Vimentin only in HT29^EMP^ cells, which was blocked by ectopic expression of CaSR (HT29^CaSR^). In HT29^CaSR^ cells, the upregulated E-Cadherin expression was downregulated upon treatment with the EMT promoting cocktail but remained higher than in HT29^EMP^ cells. The merged images (red or white channels for the indicated markers and blue for DAPI) are shown. Scale bar: 20 μm.

These results were confirmed at protein level by immunofluorescence staining (Figure 7). Treatment with the EMT promoting cocktail induced protein expression of mesenchymal markers, αSMA and Vimentin only in HT29^EMP^ cells, which was blocked by ectopic expression of CaSR. In HT29^CaSR^ cells, the upregulated E-Cadherin expression was downregulated upon treatment with the EMT promoting cocktail but remained higher than in HT29^EMP^ cells (Figure 7).

### CaSR suppresses stem cell-like phenotype in HT29 colon cancer cells

**Figure 8:**
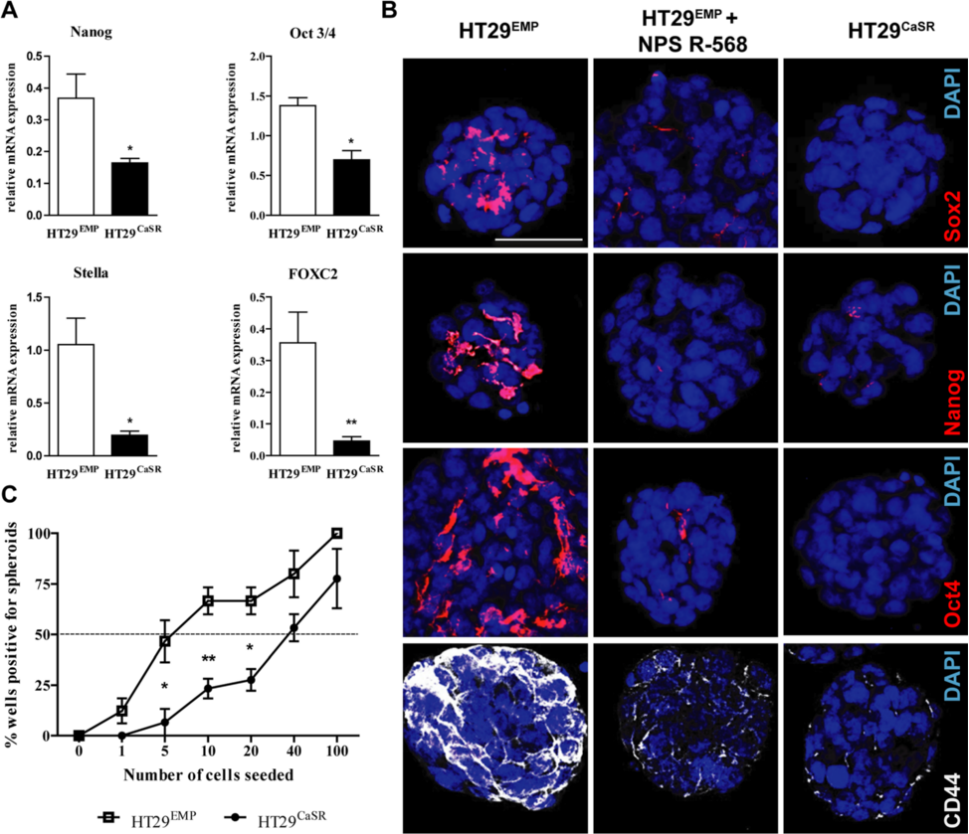
**Overexpression of CaSR blocks acquisition of stem cell-like characteristics.** HT29 cells overexpressing the CaSR (HT29^CaSR^) or empty transfected cells (HT29^EMP^) were grown in stem cell media. **(A)** Ectopic expression of CaSR blocked acquisition of the pluripotency markers Nanog, Oct3/4, Stella, and FOXC2. **(B)** Treatment with NPS R-568 or ovexpression of CaSR diminished expression of pluripotency-associated markers Sox2, Nanog, Oct4 and CD44. **(C)** Extreme limiting dilution assay revealed a significantly lower sphere forming ability of stem-like cells in HT29^CaSR^ cells. Data represent mean ± SEM of 3-5 independent experiments. Scale bar: 50 μm. Statistical significance was calculated using t test. *p < 0.05, **p < 0.01.

Since HT29 cells display expression of pluripotency-related genes, we evaluated whether expression of the CaSR could decrease the cancer stem cell-like properties of these cells. We cultured the stably transfected HT29^CaSR^ and HT29^EMP^ cells in a commercially available stem cell medium, and assessed expression of the pluripotency related genes Nanog, Oct3/4, Stella and FOXC2. mRNA expression levels of these markers was significantly lower in HT29^CaSR^ cells compared with HT29^EMP^ cells (Figure 8A).

Increasing CaSR levels, either by transfection (HT29^CaSR^) or by treatment with NPS R-568 reduced the stem-like phenotype in these cells by downregulating expression of pluripotency associated genes, SOX2, Nanog, Oct4, and the colon cancer stem cell marker, CD44 (Figure 8B).

Functionally, cancer stem cells are defined by their ability of self-renewal and form spheres *in vitro* under extreme limited dilutions [25]. Therefore, we examined whether the presence of the CaSR could block the ability of the cells to form colonospheres (*in vitro* spheroidal aggregates). When performing a limiting-dilution assay in hanging drop cultures, we found a significant difference in the spheroid forming ability between HT29^CaSR^ and HT29^EMP^ cells. While HT29^EMP^ cells needed 5 cells/drop to form spheroids in 50% of the drops (Figure 8C, open squares), HT29^CaSR^ cells required 8-fold more cells (40 cells/drop) to form spheroids in 50% of drops (Figure 8C, filled circles). Performing the Extreme Limiting Dilution Analysis (ELDA) revealed a 4-fold lower frequency (p < 0.001) in forming spheroids in HT29^CaSR^ cells (frequency: 1/60) compared with the vector-transfected control HT29^EMP^ cells (frequency: 1/15) indicating that the enrichment in these stem cell-like cells was inversely proportional to CaSR expression.

### CaSR expression positively correlates with the epithelial marker E-Cadherin, and negatively with markers of the mesenchymal lineage in human CRC samples

We evaluated *in silico* the correlation between CaSR expression and a signature of EMT markers using data available from the GEO database. This study by Ryan and colleagues [26] deposited microarray data from 111 tumor and adjacent mucosa samples from CRC patient samples.

Correlation analysis revealed a significant, negative correlation between the CaSR and markers of the mesenchymal lineage (N-Cadherin, Twist1, αSMA, Vimentin, β-Catenin, Snai2), whereas expression of the epithelial marker, E-Cadherin correlated positively with CaSR expression (Table 1).

**Table 1:** **Correlation matrix for expression of CaSR and EMT markers in human colorectal cancer samples**

	**CaSR**	**N-Cadherin**	**E-Cadherin**	**TWIST1**	**αSMA**	**Vimentin**	**β-Catenin**	**FSP1**	**SLUG**
**CaSR**	1.000	-.267^**^	.314^**^	-.212^*^	-.259^**^	-.227^*^	-.248^**^	-.152	-.397^**^
**N-Cadherin**	-.267^**^	1.000	-.157	.015	.178	.136	.100	.209^*^	.118
**E-Cadherin**	.314^**^	-.157	1.000	-.297^**^	-.230^*^	-.362^**^	-.157	-.322^**^	-.322^**^
**TWIST1**	-.212^*^	.015	-.297^**^	1.000	.193^*^	.259^**^	.101	.417^**^	.417^**^
**αSMA**	-.259^**^	.178	-.230^*^	.193^*^	1.000	.657^**^	-.054	.559^**^	.358^**^
**Vimentin**	-.227^*^	.136	-.362^**^	.259^**^	.657^**^	1.000	.077	.432^**^	.529^**^
**β-Catenin**	-.248^**^	.100	-.157	.101	-.054	.077	1.000	-.057	.256^**^
**FSP1**	-.152	.209^*^	-.322^**^	.417^**^	.559^**^	.432^**^	-.057	1.000	.408^**^
**SLUG**	-.397^**^	.118	-.322^**^	.417^**^	.358^**^	.529^**^	.256^**^	.408^**^	1.000

Correlation analysis was performed across 111 human colorectal cancer samples of tumor and adjacent non-tumor mucosa. Spearman correlation coefficient and corresponding p value is shown. **p< 0.01, *p< 0.05.

## Discussion

The calcium-sensing receptor (CaSR) has gained importance outside its physiological role as a regulator of calcium homeostasis. Several model systems have provided convincing evidence for the role of the CaSR as a tumor suppressor in the colon [18]. However, evidence describing the molecular mechanisms causing the tumor suppressive functions of the colonic CaSR is limited. In this study we demonstrate a causal relation between the CaSR and regulation of Epithelial-to-Mesenchymal Transition (EMT) as well as acquisition of stem cell-like properties *in vitro* and *in vivo*. We show that the CaSR is able to inhibit transition of colonic epithelial cells into the mesenchymal phenotype and prevents acquisition of the stem cell-like phenotype. All preliminary experiments were conducted in two colon cancer cell lines: HT29 and Caco2-15, stably overexpressing the CaSR (or the empty control vector). The results obtained from both cell lines were comparable and therefore, we focused on the HT29 cell line, which has negligible endogenous expression of CaSR.

In light of recent advances in developmental biology and biology of cancer, EMT has emerged as a critical process in the pathophysiology of inflammation and cancer [7,27,28]. Chronic inflammation in patients with inflammatory bowel disease (IBD) increases the risk of CRC development. Chemically induced, as well as genetically engineered mouse models of IBD have an increased susceptibility to develop colorectal tumors [28]. In this study, we have evaluated the expression of EMT associated markers in the colon of the exon 5-less global *CaSR/PTH* double knockout mouse as well as in the colon of the exon 7-less intestine-specific *CaSR* knockout mice. The colons of both, the global CaSR/PTH double knock out and the intestine-specific CaSR knockout mice show signs of inflammation [20,21] and immune cell activation [21] as seen also in patients with IBD [28]. We demonstrate for the first time *in vivo*, that in both models expression of EMT- associated mesenchymal markers were significantly upregulated in the colon of mice lacking the CaSR, suggesting a critical role of the receptor in suppressing EMT.

Type 2 EMT is associated with tissue regeneration and fibrosis after inflammation-associated injury. As a result, macrophages and activated fibroblasts (myofibroblasts) expressing high levels of mesenchymal markers like FSP1 and αSMA accumulate [7,29]. In some cases, epithelial cells retain a normal epithelial morphology, expressing epithelial markers like E-Cadherin but expression of mesenchymal markers is also induced [29] and cells develop into an intermediate EMT phenotype. The extent of the EMT process depends on the intensity and length of the inflammatory process as has been reported in organs like kidney, lung and intestine [7]. Eventually, these cells acquire mesenchymal characteristics, lose cell-cell contact, migrate out of the epithelial layer and enter the interstitium, where they forego their epithelial phenotype and attain a mesenchymal phenotype [30]. Therefore it is not surprising, that we observe enrichment in populations of stromal cells staining positive for expression of mesenchymal markers in the colon of the CaSR^int-KO^ mice. In the colon of these mice, the cadherin-switch leads to a significant upregulation of N-Cadherin expression without significant alterations in E-Cadherin levels. Such cadherin switch has been previously reported in other cancers [12,31].

The enhanced inflammatory/immune cell microenvironment can eventually lead to cancer through the inflammation-dysplasia-carcinoma sequence. Extracellular calcium is known to have tumor preventing effects in colorectal cancer [32] and these effects are mediated by the CaSR [18,22,23]. CaSR-null cells, which represent a subpopulation of colon cancer cells, indeed show an enhanced malignant phenotype with increased migration potential and enhanced expression of EMT markers [33].

Several colon cancer cell lines of epithelial origin, including the HT29 cells used in this study, are reprogrammed to acquire an EMT-like phenotype (mesenchymal-like morphology, loss of cell-cell adherence and a highly metastatic and invasive phenotype). These cell lines have low endogenous CaSR expression compared with more differentiated colon cancer cells having a more epithelial phenotype (e.g. Caco-2 cells) [34]. In normally functioning colon cells, β-catenin (a member of the canonical Wnt pathway) binds to E-cadherin and is involved in cell-cell adhesion. Free cytosolic β-catenin is marked for degradation in the proteasome by the APC/Axin/GSK-3β destruction complex. However, when the Wnt pathway is activated, cytosolic β-catenin is translocated to the nucleus where it binds to the Transcription Factor (Tcf)-4 and regulates transcription of genes involved in proliferation, differentiation and apoptosis [11,35-39]. Active Wnt signaling can prevent GSK-3β induced degradation of Snail, and thereby promote EMT [40].

In the present study we show that reintroduction of the CaSR induced a transformation in the morphological architecture of HT29 cells from a more spindle-like, fibroblast-like phenotype to a well adherent, epithelial-like phenotype. These cells also showed reduced cellular invasiveness and migration, two critical steps for initiation of metastasis. Furthermore, HT29 cells overexpressing the CaSR had increased GSK-3β and E-Cadherin expression, in parallel with reduced nuclear β-catenin and Cyclin D1 levels. These data support previous findings that the CaSR suppresses the malignant behavior of CRC cells by modulating the Wnt signaling pathway [20], which is often deregulated in CRC. Rey and colleagues have previously shown the CaSR-dependent regulation of Wnt/β-catenin pathway in a normal colonic epithelial cell line with a functional APC gene [41]. It is interesting that although HT29 cells harbor a mutation in the APC gene, overexpression of the CaSR is able to counteract defective β-catenin activity in these cells.

Singh and colleagues have recently observed an EMT-driven, highly malignant phenotype in CaSR-null cells [33]. However, it remained elusive to what extent the CaSR is able to regulate/interfere in the EMT process. By treating stably transfected HT29 cells with an EMT inducing supplement, we are the first to report that the CaSR is effective in reversing the EMT phenotype and that the presence of the CaSR effectively blocks the transition from an epithelial to a mesenchymal state even under EMT promoting conditions. We further show that treatment with the calcimimetic, NPS R-568 previously described as a pharmacochaperone of the CaSR [42], was able to induce endogenous CaSR expression. Both, ectopic overexpression and enhancement of endogenous CaSR expression and activity (treatment with NPS R-568) is able to reverse the EMT phenotype by upregulating expression of E-Cadherin, which becomes localized to the cell membrane, and by downregulating expression of EMT-associated mesenchymal markers.

In several cancers, acquisition of EMT characteristics leads to a parallel increase in pluripotency-associated markers like Nanog, Stella and Oct3/4 [43,44]. In solid tumors (like the colon), these subpopulations of cells, which express cell surface markers like CD44, are termed cancer stem-like cells. These cancer stem cells, although controversial, have attained a lot of research interest recently for their contribution to recurrence/relapse of chemoresistant tumors [45]. HT29 cells express cancer stem cell markers abundantly. Since reintroduction of the CaSR induces a more differentiated phenotype, we predicted a reduction in their stem-like phenotype as well. Culturing colonospheres (*in vitro* spheroids) of stably transfected HT29^EMP^ and HT29^CaSR^ cells in chemically defined media supporting growth of stem cells [46], we were able to show that the CaSR was not only able to downregulate expression of markers associated with stemness, but was also able to reduce the ability to form tumor-associated spheroids.

## Conclusions

Our data demonstrates that reintroduction of the CaSR prevents the development of a highly malignant, stem cell-like phenotype in colon cancer cells. The mechanisms involved in this process advance our understanding of the molecular changes in colon cancer cells accompanying the loss of CaSR. We show that the CaSR is necessary for calcium-mediated growth control in the colon. These data support the rationale to develop pharmaceutical agents to restore expression and function of colonic CaSR during colonic inflammation and cancer.

## Methods

### *CaSR/PTH* double-knockout mouse *(CaSR*^−/−^/*PTH*^−/−^)

Mice heterozygous for *CaSR*^ΔExon5^ and *PTH* were bred to generate *CaSR*^+/+^/*PTH*^−/−^ and *CaSR*^−/−^/*PTH*^−/−^ mice as previously described [47]. All mice were maintained under standard conditions as approved by the Institutional Animal Care and Use Committee at Harvard Medical School. Age- and sex-matched animals (n = 9/genotype) were sacrificed; colons were washed in ice-cold PBS and stored in RNAlater (Life Technologies, Austria). For mRNA analysis, 1–2 cm of colonic tissue, 0.5 cm distal from cecum was used.

### Intestine specific CaSR knockout mouse (CaSR^int-KO^)

CaSR^flox/flox^ mice [48] were bred with mice genetically engineered to express Cre-recombinase under the control of the villin 1 promoter to produce ^vil^Cre/CaSR^flox/flox^ and CaSR^flox/flox^ mice, as previously described [41]. All mice were maintained under standard conditions as approved by the Animal Care Subcommittee at San Francisco Department of Veterans Affairs Medical Center. Age- and sex-matched animals (n = 5/genotype) were sacrificed, whole colons were washed in ice cold PBS, fixed and embedded in paraffin until further analysis.

### Cell culture, cloning and stable transfection

The human colon cancer cell line HT29 was obtained from American Type Culture Collection (ATCC, USA) and was routinely maintained in Dulbecco’s Modified Eagle Medium (DMEM) containing 10% FBS, 1.8 mM Ca^2+^, 2 mM L-glutamine, 100 U/ml penicillin, and 100 μg/ml streptomycin (all from Life Technologies) in a 5% CO_2_/humidified air incubator maintained at 37°C. Cells were periodically tested for mycoplasma contamination and authenticated by STR DNA profiling (DNA Diagnostic Center, UK).

HT29 cells were transfected with pcDNA3.1/Zeo^(+)^ (EMP) or an expression vector encoding the full length CaSR cDNA (constructs kindly provided by Prof. Romuald Mentaverri, University of Picardie Jules Verne, France) using Lipofectamine LTX reagent (Life Technologies) as previously described [18]. Stable transfectants were selected by culturing the cells in the presence of Zeocin (150 μg/ml) for over 6 months.

### Invasion assay

*In vitro* cellular invasion was determined using the Cultrex^®^ 3D Spheroid Cell Invasion Assay (Trevigen, USA) according to the manufacturer’s instructions. Briefly, cells were seeded at a density of 3000 cells/cm^2^ (in triplicates) in 96-well spheroid forming plates along with an extracellular matrix to drive aggregation of spheroids. After 72 hours, the spheroids were allowed to invade into a matrix composed of basement membrane proteins. Cellular invasion was visualized with a bright field microscope and quantified using Adobe Illustrator (Adobe Systems, USA) to calculate the area of the invading spheroids (invasive index) and the number of invading daughter spheroids. The assay was performed in three independent experiments.

### Nuclear and cytosolic protein extraction and western blot analysis

In order to retrieve nuclear and cytosolic protein fractions, cells were cultured to 60-70% confluency, collected in ice-cold PBS and centrifuged. The pellet was first resuspended for 15 minutes in a hypotonic buffer (10 mM Hepes pH 7.5, 10 mM KCl, 0.1 mM EDTA, 0.1 mM EGTA, DTT, PMSF, protease and phosphatase inhibitors and 10% NP40 (all from Sigma Aldrich, Germany) and centrifuged to obtain the soluble cytosolic fraction in the supernatant. Next, the pellet was resuspended in a high salt buffer (20 mM Hepes pH 7.5, 0.4 M NaCl, 1 mM EDTA, 1 mM EGTA, DTT, PMSF, protease and phosphatase inhibitors) for 15 minutes to obtain the soluble nuclear fraction by centrifugation. Protein concentration was measured using Protein Assay Dye (Bio-Rad, USA).

Equal amounts of protein lysate were separated using sodium dodecyl sulfate polyacrylamide gel electrophoresis and transferred to a nitrocellulose membrane. Membranes were blocked in 5% milk (in 10 mM Tris, pH 7.5, 150 mM NaCl, 0.1% Tween-20 (TBST)) for 1 h at room temperature (rt) and subsequently incubated with the primary antibodies (in TBST) for 1 h at rt. After washing, the membrane was incubated with respective secondary antibody for 1 h at rt. The membrane was subjected to ECL reagent (Bio-Rad), and protein bands were detected using a digital imaging system (VersaDoc) and quantified using Image Lab software (Bio-Rad).

Primary antibodies used were β-Catenin (1:5000, Abcam, UK), α-Tubulin (1:10000, Sigma Aldrich), Lamin C (1:1000, Santa Cruz Biotechnology Inc, USA). Secondary antibodies used were horseradish-peroxidase-coupled anti-mouse (1:5000, Jackson ImmunoResearch, UK), anti-rabbit (1:10000, Jackson ImmunoResearch) and anti-goat (1:20000, AbD Serotec, UK) IgG.

### Induction of EMT

Stably transfected cells were treated with a commercially available EMT-inducing cocktail for HT29 cells according to the manufacturer’s instructions (R&D Systems, USA) [49]. Cells were cultured in DMEM media containing 5% FCS and 1.8 mM Ca^2+^ in the presence or absence of 1X EMT inducing supplement for 6 days and data analyzed for mRNA/protein expression.

### Induction of stem cell-like phenotype

Stably transfected cells were cultured in the commercially available Essential 8 stem-cell media [46] according to the manufacturer’s instructions (Life Technologies). Cells were cultured for 6 days and analyzed for mRNA/protein expression and colonosphere-forming ability. For protein expression studies, stably transfected cells were also treated with 1 μM NPS R-568 (in DMSO). Vehicle-treated cells were used as controls.

For the colonosphere-forming assay we modified the extreme limiting dilution analysis (ELDA) by Yu et al. [50] originally described by Hu and Smyth [51]. Cells were trypsinized to obtain single-cell suspension and were then seeded at the concentrations of 100, 40, 20, 10, 5, and 1 cell(s) per 50 μl Essential 8 media (6 replicates for each dilution/run) as hanging drop cultures. One week post seeding, the percentage of wells positive for formation of colonospheres was determined and plotted against the number of cells seeded per drop. Sphere forming frequency was determined using the ELDA analysis tool at http://bioinf.wehi.edu.au/software/elda.

### RNA isolation, reverse transcription and quantitative RT-PCR

Total RNA was isolated using Trizol reagent (Life Technologies) according to the manufacturer’s instructions. Integrity of RNA was checked by agarose gel electrophoresis, and RNA was reverse transcribed as previously described [52]. qRT-PCR was performed on the Step One Plus qRT-PCR system using Power SYBR Green master mix (Life Technologies). Where possible, primers were designed to bridge an exon-exon junction to prevent genomic DNA from being amplified. The ΔΔC_t_ method was used to calculate fold changes in gene expression, relative to housekeeping genes and normalized to a commercially available total RNA calibrator (Clontech, USA) according to Livak et al. [53].

Human Beta-actin (hβ-ACTIN), human Large ribosomal protein (hRPLPO) and/or human Beta-2-microglobulin (hβ2M) were used as housekeeping genes for samples of human origin; mouse β-actin and mouse Eukaryotic translation elongation factor 1 beta 2 (mEef1B2) were used as housekeeping genes for mouse colon samples. Primer sequences are shown in Additional file 1: Table S1.

### Immunostaining

Cells grown on glass cover slips under experimental conditions as well as paraffin-embedded 5-μm tissue sections were stained as previously described [17]. Samples were incubated with primary antibodies for 1 h at rt. Isotype-specific IgG antibodies were used as negative controls. Samples were subsequently incubated with corresponding secondary antibodies for a further 1 h at rt. Nuclei were stained with DAPI (1:3000, Roche, Switzerland) and images acquired with the TissueFAXS system (TissueGnostics, Austria).

Primary antibodies used were, anti-E-Cadherin (1:200), anti-Snai1 (1:100), anti-Snai2 (1:200) and anti-Vimentin (1:100), (EMT Antibody Kit, Cell Signaling Technologies, Austria) and anti-Sox2, anti-Nanog, anti-Oct4 (all 1:400, StemLight™ Pluripotency Transcription Factor Antibody Kit, Cell Signaling Technologies). Anti-CaSR (1:200) and anti-CD44 (1:100) antibodies were purchased from Abcam and the anti-αSMA (1:100) was purchased from Sigma Aldrich. Secondary antibodies used were: Alexa Fluor-647 goat-anti-mouse (1:1,000, Life Technologies) or Dylight labeled-549 goat-anti-rabbit antibody (1:500, Vector Laboratories).

### Acquisition and processing of public microarray data

Raw microarray data was obtained from the Gene Expression Omnibus (GEO, http://www.ncbi.nlm.nih.gov/geo) database. The dataset GSE44861 deposited by Ryan and colleagues contained log2 transformed expression data from 111 tumor and adjacent non-tumor samples from CRC cancer patients [26] and was used to compute the correlation between the CaSR and EMT markers studied.

### Statistical analysis

All assays were performed in at least three independent experiments. For comparison between 2 groups, t-test was used. For group comparisons, Analysis of Variance (ANOVA) was performed followed by Tukey’s post-test. Non-normally distributed data were log-transformed to achieve normal distribution. Correlation coefficients were calculated using the nonparametric Spearman’s correlation. P values <0.05 were considered statistically significant. SPSS (IBM, USA) was used to perform all statistical calculations and graphs were plotted using GraphPad Prism (GraphPad Software Inc., USA).

## Additional file

Additional file 1: Table S1. Details of primers used in the study.

## Abbreviations

αSMA: alpha smooth muscle actin
ANOVA: Analysis of variance
CaSR: Calcium-sensing receptor
CDX2: Caudal type homeobox 2
CRC: Colorectal cancer
ELDA: extreme limiting dilution analysis
EMT: Epithelial-to-mesenchymal transition
FOXC2: Forkhead box protein C2
FSP: Fibroblast specific protein
IBD: Inflammatory bowel disease
MET: Mesenchymal-to-epithelial transition
PTH: Parathyroid hormone
Snail: Snai1
Slug: Snai2
TCF4: Transcription factor 4
ZEB: Zinc finger E-box binding
5-FU: 5- fluorouracil
