# Supplementary material for: The calcium-sensing receptor suppresses epithelial-to-mesenchymal transition and stem cell- like phenotype in the colon
Source: Mol Cancer. 2015 Mar 18;14:61. doi: 10.1186/s12943-015-0330-4 (PMC4405849; doi:10.1186/s12943-015-0330-4)
Supplement: Additional file 1: Table S1. — Details of primers used in the study. [file 12943_2015_330_MOESM1_ESM.docx]

|  | Forward | Reverse |
| --- | --- | --- |
| HUMAN |  |  |
| CDX2 | AGGGGGTGGTTATTGGACTC | CATTCAGCCCAGAGAAGCTC |
| Cyclin D1 | GAACAAGCTCAAGTGGAACC | GAACTTCACATCTGTGGCA |
| Villin | AACACCAAGAGACTACAGGA | CACATCATCCTCTTCCAAGTC |
| E-Cadherin | GCACAGCCTGTCGAAGCA | GGGCAGTAAGGGCTCTTTGA |
| GSK-3β | TTGGACTAAGGATTCGTCAGG | TGTTAGTCGGGCAGTTGGT |
| αSMA | CACCATCGGAAATGAACGTTT | GACTCCATCCCGATGAAGGA |
| SNAI1 | CGCTACTGCTGCGCGAAT | GCTGGAAGGTAAACTCTGGATTAGA |
| Twist2 | GCCAGGAGGAGATTCTGAATGA | TTCAAGCATGTTCTTAGCCATTG |
| Vimentin | GAAGAAACTCCACGAAGAGGAAAT | GCTTGGAAACATCCACATCGA |
| Zeb1 | TCACATAAATCAGGAAGAGATCAAAGA | AGCTTTTCCACACTCAGTGCATT |
| Nanog | ATGCCTCACACGGAGACTGTCT | TGACCGGGACCTTGTCTTCC |
| Stella | GCGGAGTTCGTACGCATGA | CGCAGAAACTGCAGGGACAT |
| Oct3/4 | CAACCTGGAGAATTTGTTCCT | CTCGGACCACATCCTTCTC |
| β-Actin | TGGCTCCCGAGGAGCAC | TTGAAGGTCTCAAACATGAT |
| β2M | GATGAGTATGCCTGCCGTGTG | CAATCCAAATGCGGCATCT |
| RPLPO | CCTCATATCCGGGGGAATGTG | GCAGCAGCTGGCACCTTATTG |
| hFOXC2 was analyzed using TaqMan probe (Hs00270951_s1) | | |
|  | | |
| MOUSE |  |  |
| Fsp1 | TCTTGGTCTGGTCTCAACGG | GCCTGAGTATTTGTGGAAGGT |
| Slug | GGCTGCTTCAAGGACACATT | TTGGAGCAGTTTTTGCACTG |
| Zeb1 | AGCAGACCAGACAGTATTACC | CTCGTTGTCTTTCACGTTGTC |
| Vimentin | CAAGTCCAAGTTTGCTGACC | CTTTAAGGGCATCCACTTCAC |
| Twist2 | GCAAGAAGTCGAGCGAAGAT | GCTCTGCAGCTCCTCGAA |
| Nanog | TTGCTTACAAGGGTCTGCTACT | ACTGGTAGAAGAATCAGGGCT |
| Stella | AGACTTGTTCGGATTGAGCAG | ATAATGGCAGAAAGTGCAGAG |
| Eef1B2 | AGAGCTACATTGAGGGGTACG | GACTTGATGTGATTATACCAACGTAG |
| β-actin | TCCTAGCACCATGAAGATCA | CCACCGATCCACACAGAGTA |
| mαSMA was analyzed using TaqMan probe (Mm00725412_s1)) | | |

**Supplementary Table S1.** Details of primers used in the study.
